# Projective oblique plane structured illumination microscopy

**DOI:** 10.1038/s44303-023-00002-2

**Published:** 2023-11-28

**Authors:** Bo-Jui Chang, Douglas Shepherd, Reto Fiolka

**Affiliations:** 1 Lyda Hill Department of Bioinformatics, University of Texas Southwestern Medical Center, Dallas, TX 75390 USA; 2 Center for Biological Physics and Department of Physics, Arizona State University, Tempe, AZ 82587 USA

**Keywords:** Light-sheet microscopy, Super-resolution microscopy

## Abstract

Structured illumination microscopy (SIM) can double the spatial resolution of a fluorescence microscope and video rate live cell imaging in a two-dimensional format has been demonstrated. However, rapid implementations of 2D SIM typically only cover a narrow slice of the sample immediately at the coverslip, with most of the cellular volume out of reach. Here, we implement oblique plane structured illumination microscopy (OPSIM) in a projection format to rapidly image an entire cell in a 2D SIM framework. As no mechanical scanning of the sample or objective is involved, this technique has the potential for rapid projection imaging with doubled resolution. We characterize the spatial resolution with fluorescent nanospheres, compare projection and 3D imaging using OPSIM and image mitochondria and ER dynamics across an entire cell at up to 2.7 Hz. To our knowledge, this represents the fastest whole cell SIM imaging to date.

## Introduction

Structured illumination microscopy (SIM) can double the resolution of a fluorescence microscope, is compatible with commonly used fluorophores and sample preparations, requires only modest to medium laser powers and is fast enough to follow cellular dynamics^1,2^. As such, it has established itself as an important tool in biological fluorescence microscopy. However, the fastest SIM acquisition rates, up to several tens of Hz, have been achieved in 2D SIM, typically in Total Internal Reflection Fluorescence (TIRF) or grazing incidence (GI) modalities to improve optical sectioning^3–6^. Physical background suppression is necessary, as 2D SIM can be prone to artifacts from out-of-focus blur. As such, imaging is locked to the coverslip-cell interface. While this enables rapid and sensitive imaging of the plasma membrane, it does not allow deeper imaging inside the cell or for example to visualize the dorsal membrane. 3D SIM breaks free from the coverslip for volumetric acquisitions at the cost of acquisition speed, as 15 stacks of different illumination phases and orientations need to be acquired^7,8^. Processing of a single slice at an arbitrary focal plane has been suggested theoretically^9^ and implemented practically^10,11^, and as such can reach faster rates than 3D SIM. However, it then only covers a slice limited by the microscope’s depth of focus (<1 μm) and suffers from more background and related artifacts compared to TIRF or GI-SIM.

Here, we report projection imaging under structured illumination to image an entire cell at acquisition rates faster than 1 Hz. We leverage two recent innovations, oblique plane structured illumination microscopy (OPSIM)^12^ and multi-angle projection imaging^13^. Projective OPSIM, or POPSIM for short, enables 2D SIM imaging of projections of an entire cell instead of a thin slice. Because POPSIM projects the volume under structured illumination into a 2D plane, only nine raw images are required to reconstruct the projected volume. As such, rapid organelle dynamics can be captured throughout a cell, and imaging is not tied to the coverslip-cell interface. Thus, for many rapid dynamics, and processes for which simultaneous observation is paramount, projection imaging represents a valuable alternative to slower 3D stacking.

**Fig. 1:**
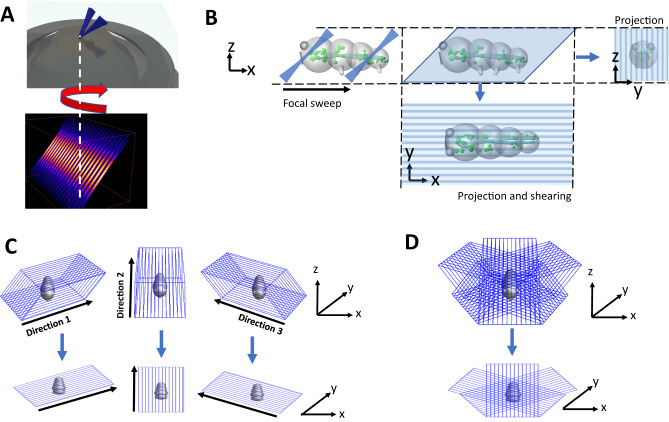
Schematic representation of the POPSIM concept. **A** In OPSIM, a structured light sheet (numerical simulation of the intensity pattern shown below) emerges from a high numerical aperture lens. The structured light sheet can be azimuthally rotated, phase stepped, and scanned laterally to acquire stacks. **B** Schematic illustration of projection imaging. The light-sheet and focal plane are rapidly swept through the sample, covering the sample (light-blue parallelogram). If at least one sweep occurs during a camera exposure, a sum projection is formed (right). By synchronously shearing the images on the camera during the focal sweep, a projection along the z-axis can be formed (bottom). **C** In OPSIM stacks under different phases of the structured light-sheet are acquired for three azimuthal directions (top). In POPSIM, each volume is projected along the z-axis (bottom). **D** In OPSIM, volumes acquired under different directions are computationally registered in 3D to each other. In POPSIM, the corresponding projections are registered in 2D to each other.

The concept for POPSIM is schematically illustrated in Fig. 1. It leverages OPSIM, which combines light-sheet microscopy and structured illumination in a single objective format^12,14^ (Fig. 1A). To this end, two mutually coherent light-sheets interfere in a common oblique plane which is inclined to the optical axis. The light sheets can be rotated, phase-stepped and scanned laterally. The OPSIM architecture is compatible with our recently introduced multi-angle projection technique:^13^ by rapidly sweeping the structured light sheet through the sample, and optically shearing the resulting image over the camera detector, a sum projection of the sample is created optically via the shear-warp transform^15^, as schematically shown in Fig. 1B. As such, a projection of a volume under structured illumination can be formed optically.

For a full OPSIM reconstruction, three stacks under different phases of the illumination pattern were acquired for three azimuthal orientations of the illumination and detection path. We reasoned that if all nine volumes in OPSIM are projected into the same coordinate frame, then SIM processing in a conventional 2D format can be performed. This idea is schematically shown in Fig. 1C: volumes that are spanned by rapidly sweeping the structured light sheet are projected in a “top down” view, i.e., along the normal direction to the coverslip surface (labeled z). The three illumination directions (labeled “Direction 1–3”, also called “SIM orientations” henceforth) are mapped into the lateral *x*-*y* plane.

In OPSIM, volumes from the three illumination directions need to be 3D registered to each other. In the projective variant, the registration happens in 2D, as schematically shown in Fig. 1D. Consequently, each projection needs to be aligned precisely along the z-direction for each illumination direction, else sample features from different depths cannot be overlapped by subsequent 2D registration (Supplementary Note 1 and Supplementary Fig. 1). We have developed a calibration procedure for the projection parameters which is discussed in the Methods section and Supplementary Fig. 2.

In contrast to traditional OPSIM, where hundreds of image frames need to be acquired to span a single volume, acquiring only nine projection images in POPSIM promises a significant reduction in data volume and a corresponding improvement in acquisition speed. Compared to other rapid 2D SIM modalities, POPSIM extends the volumetric coverage 10–100-fold (assuming a cell of ~10 μm height). Being a light-sheet-based technique, it also provides a different mechanism for out-of-focus suppression that is not bound to the coverslip, compared to TIRF or GI-based illumination schemes.

For projection imaging in general, contrast and resolution are often compromised compared to 3D imaging modalities. Here, we show that structured illumination can provide a mechanism to improve resolving power and contrast. While our implementation of projection imaging is specific to a class of light-sheet microscopes, we think our results using structured illumination can be generalized to other projection modalities that are recorded in a widefield format^16–19^.

## Results

### Resolution measurements

**Fig. 2:**
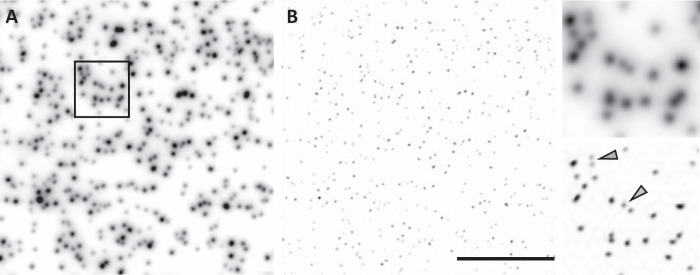
Resolution measurements using fluorescent nanospheres. **A** Projection image of 100 nm fluorescent nanospheres obtained by oblique plane microscopy. **B** POPSIM reconstruction of the same bead dataset. Insets show magnified regions of the black-boxed area shown in (**A**). Chevrons point at closely spaced beads that are resolved by POPSIM, but not by the conventional projection image. Scale Bar: 10 μm.

We evaluated the resolution of POPSIM by imaging 100 nm fluorescent nanospheres (Polysciences, Inc.). Figure 2A shows a representative projection view in conventional oblique plane microscopy (OPM). Figure 2B shows the same area, but after a SIM reconstruction of the POPSIM data. Chevrons point at pairs of fluorescent nanospheres that were resolved by structured illumination but cannot be readily distinguished in the conventional projection image. We estimated the resolution by measuring the full width half maximum (FWHM) over multiple beads. In Fig. 2A, the FWHM was 381 ± 44 nm and 386 ± 29 nm (mean and standard deviation, *n* = 22) in the *x*- and *y*- direction, respectively. This is a lower spatial resolution than what the same microscope can produce in a 3D imaging modality, likely because the 3D PSF is summed along the z-direction: the sum projection involves adding portions that are slightly outside of the depth of focus of the imaging system, resulting in slight blurring of the projection PSF. This is in part also due to the axially narrow detection PSF compared to the thickness of the light sheet.

Accordingly, we set the linespacing of the structured light sheet to ~390 nm, resulting in a frequency that is of similar magnitude as the cut-off frequency of our projection OTF. This linespacing is a bit wider than what practically is possible on our setup (~300 nm at 488 nm excitation wavelength). In the SIM mode, we measured an FWHM of 185 ± 10 nm and 189 ± 11 nm (*n* = 22) for the x- and y-direction, respectively, using a traditional SIM reconstruction algorithm with Wiener filtering^20^. Replacing Wiener Filtering with five iterations of Richardson Lucy (RL) deconvolution resulted in an FWHM of 161 ± 11 nm and 169 ± 9 nm (*n* = 22), respectively, while image decorrelation analysis^21^ estimated a resolution of 178 nm. The RL deconvolution was not used to boost the resolution further. Instead, we found that the RL-assisted reconstruction was more robust than the classical SIM algorithm: there was little to no parameter tuning necessary and it also reduces background haze, which can accumulate due to the sum projection in more densely labeled samples (a more detailed discussion can be found in previous work^12,22^. For the data presented here, we used the same code as for the slice by slice reconstruction in OPSIM^12^). As such, we employed the RL-assisted reconstructions of the biological imaging shown below.

### Cellular imaging of fixed cells

**Fig. 3:**
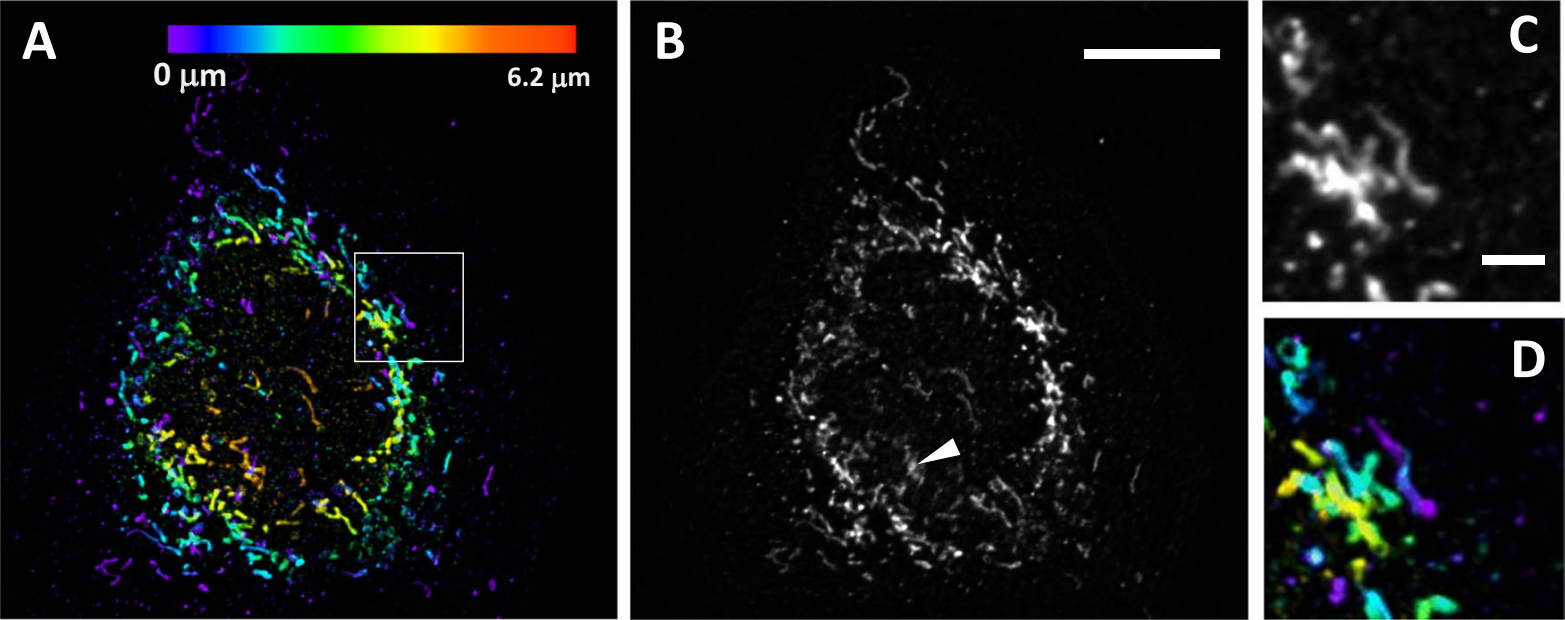
Comparison of 3D and projective imaging. **A** An U2OS cell labeled with MIC60, as imaged with OPSIM. Depth is color coded. **B** The same cell as imaged under POPSIM. The white Chevron points at a reconstruction artifact. **C**, **D** Magnified views of the boxed region in (**A**), under POPSIM (**C**) and OPSIM (**D**). Scale bar: 10 μm.

To compare volumetric and projective SIM modalities, we imaged a fixed U2OS cell where mitochondria were stained with Alexa 488 using antibody labeling for MIC60. We further leveraged the fact that we can perform OPSIM and POPSIM imaging on the same setup. In Fig. 3A, a maximum projection image, color-coded for depth, of the OPSIM data is shown. Notably, mitochondria are present at different heights, with two mitochondria in the center being the highest above the coverslip. In Fig. 3B, the corresponding POPSIM image is shown. The projection encompasses the entire cell, so mitochondria near the coverslip as well as the mitochondria presumably above the nucleus are captured. The insets in Fig. 3C, D show a comparison between 3D and projective imaging. Similar morphological detail is captured of a cluster of mitochondria. Generally, there is good correspondence between features and morphologies, with some exceptions (see for example the chevron in Fig. 3B where the reconstruction of mitochondria appears blurrier in the POPSIM image).

### Dynamic live cell imaging

**Fig. 4:**
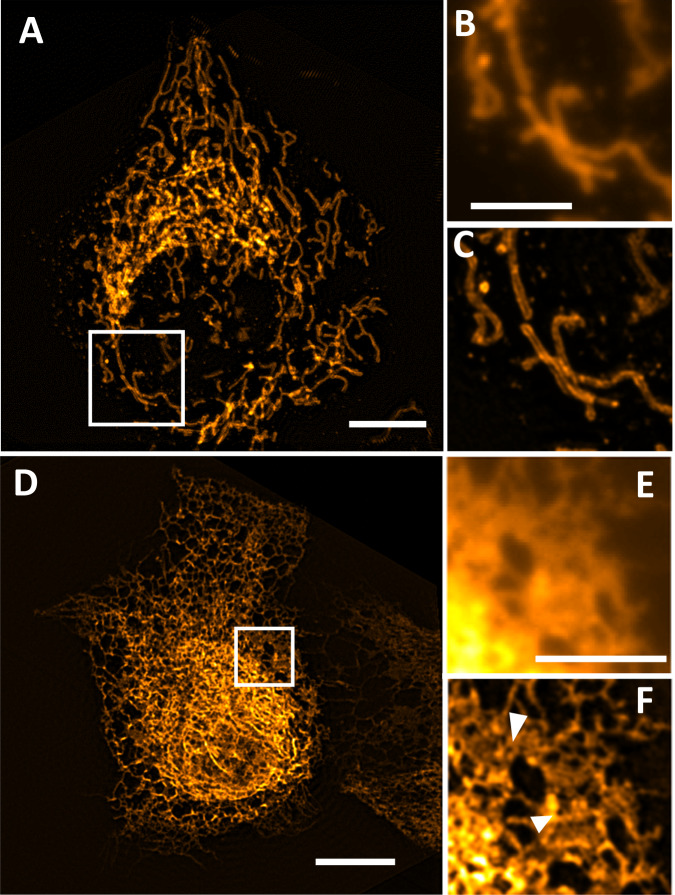
Live imaging with POPSIM. **A** U2OS cells labeled with OMP-GFP, as imaged with POPSIM at 2.2 Hz overall rate. A two-frame average is shown. **B** Magnified view of the boxed region in (**A**) as imaged with projection OPM. **C** Magnified view of the boxed region in (**A**) as imaged with POPSIM. **D** An U2OS cells expressing the endoplasmic reticulum label Sec61-GFP, as imaged by POPSIM at 2.7 Hz overall rate. A two-frame average is shown. **E** Magnified view of the boxed region in (**D**), as imaged with projection OPM. **F** Magnified view of the boxed region in (**D**) as imaged with POPSIM. Chevrons point to small holes in ER sheet. Scale bars: **A**, **D**: 10 μm **B**, **E**: 5 μm.

We explored the temporal capabilities of POPSIM by imaging live U2OS cells labeled with OMP-GFP, an outer membrane marker of mitochondria, at a rate of 2.2 Hz (Fig. 4A–C). Subsequent frames showed some jitter artifacts, which may originate from fluctuating edge artifacts the camera exhibited at this speed, or rapid motion of the sample itself. By taking a two-frame average, the artifacts mostly disappeared. The magnified views reveal that POPSIM was able to resolve the hollow structure of the mitochondria (Fig. 4C). Since POPSIM performs a sum projection along the z-axis, we expect that the mitochondria walls are less sharply resolved as in a 3D SIM modality, where either a cross-section or a maximum intensity projection can be shown. In Supplementary Movie 1, mitochondria dynamics over 100 timepoints are shown. In multiple instances, rapid protrusion and retraction of mitochondria could be observed.

We next imaged live U2OS cells expressing Sec61-GFP, a label for the endoplasmic reticulum (ER) at a rate of 2.7 Hz (Fig. 4D–F). POPSIM was able to follow rapid rearrangements of the ER (Supplementary Movie 2). We were also able to resolve fine structures in ER sheets that may represent nanoholes that have been recently described with rapid 2D super-resolution methods^23,24^. These holes persisted in some instances over multiple frames and appeared to move along the sheet. Of note in Fig. 4D, the top right corner appears darker than the rest of the image. This edge marks the end of scan direction 3, and the image registration algorithm filled the corner with zeros. Similarly, we believe that edge artifacts may also be the reason for some stripe artifacts in Fig. 4A.

## Conclusions

In summary, we have introduced a rapid way to image entire cells in a projective format with structured illumination. This extends the capabilities of structured illumination to rapidly capture dynamics across an entire cell at rates that cannot be achieved with 3D super-resolution methods. Compared to other rapid 2D SIM methods, which can only capture a thin slice of 100–1000 nm thickness, POPSIM can capture organelle dynamics in a projection of an entire cell. As our imaging was performed on cells with about 10 μm height, a ten- to hundredfold larger volume is sampled than in TIRF- or GI-SIM. This is, in our view, an important distinction and represents complementary capabilities to other SIM and super-resolution modalities.

Our POPSIM instrument is based on an oblique plane microscope equipped with an NA 1.35 silicone oil objective. As such, it is best suited to single-cell imaging, due to the limited range over which remote focusing, a key component of OPM, is diffraction limited. Nevertheless, we believe that POPSIM could also be implemented with NA 1.1–1.2 water immersion objectives which possess longer working distances. A separate constraint is how well the shear warp projections map the three SIM orientations into the same plane. This depends on how well the shear unit is calibrated, and how well OPM performs telecentric scanning of a volume.

Simpler implementations of POPSIM with only one SIM direction could be envisioned. This would dispense with the shear calibration and image registration, and light-sheet systems with one illumination direction could be used^25,26^. Further, it would enable arbitrary viewing directions, compared to the fixed “top down” view employed here, at the expense of anisotropic resolution. Coarser patterns could be applied in this context to increase the optical sectioning strength^27,28^, which could be valuable in the presence of large background levels.

3D reconstructions could conceivably be retrieved from POPSIM data when for each SIM direction multiple viewing angles were acquired. Such a scheme may still provide a speed advantage over conventional 3D stacking. Here, more complex structured illumination patterns, combined with the different views of the sample, could improve the overall 3D reconstruction. In contrast to the top-down view, the axial resolution of the system would be of importance. We assume that the upper limit of axial resolution would be bounded by the axial resolving power of a conventional, 3D stacking implementation of OPSIM.

The field of view was limited to about 60 × 60 μm^2^ by the image rotator used in our OPSIM instrument^12^. An optimized rotator design and lager galvo mirrors could increase the field of view. However, larger galvos would also lead to a slower switching time. An alternative approach would be to move the rotator into a reciprocal space, i.e., close to a plane conjugate to the pupil of the primary objective. This could decouple the mirror size from the achievable field of view.

Acquisition speed was in our first experiments mainly limited by hardware control, not sample brightness and camera rate (see also Supplementary Note 2 for the camera frame rate limits). The projection imaging itself is able to match the maximum frame rate of our camera (~200 frames/s) using fast galvo scanners^13^. While phase stepping is possible on millisecond timescales with piezo actuators^29^, or faster with electro optical modulators^30^, the slowest step is image rotation, due to the relatively large rotation angle of the galvos. This occurs though only for every third image and may add ~6 ms per SIM timepoint. We estimate that with current technology, POPSIM imaging at 16 Hz overall framerate could be possible.

Another important aspect of POPSIM is the analog summation of photons and reduction of read noise compared to digital projections of 3D data sets. This can intuitively be explained as follows: if the same volume were to be acquired by OPSIM, many more image frames at short exposure time need to be acquired, which in turn accumulate more camera-induced noise when forming a digital projection of the data. We have performed simulations in Supplementary Note 3, which suggests that POPSIM may be advantageous in low photon count imaging regimes.

The resolution of POPSIM is lower than what SIM systems using sinusoidal interference patterns can achieve. As an example, TIRF-SIM can achieve resolution levels slightly below 100 nm^31^. This is in part due to the twice as fine line spacing of the interference patterns (~185 nm compared to 390 nm used here, assuming 488 nm excitation wavelength) that can be generated in evanescent fields, and the smaller detection PSF. Finer line spacings could be generated in POPSIM using higher NA TIRF objectives. Such oil immersion objectives can potentially be used for OPM-based 3D imaging in watery samples, owing to new insights into remote focusing^32^. Such adjustments of the OPM system to the sample’s refractive index may also enable larger volumetric coverage, up to the limits of remote focusing theory^33^.

Since the only moving parts are of low inertia (galvo mirrors), POPSIM has the potential for high-speed imaging that is camera or fluorescence limited. It could advantageously be combined with TIRF- or GI-SIM in a “smart” microscopy format^34^. As such, clathrin-mediated endocytosis could be monitored at the plasma membrane together with intracellular transport, just to name one application of such a hybrid modality.

We anticipate that POPSIM adds new spatiotemporal imaging capabilities to fluorescence microscopy, which may prove useful to follow rapid cellular dynamics, and may spur further developments in OPM, SIM, and projection imaging.

## Methods

### Experimental setup

For the first demonstration of POPSIM presented in this manuscript, we added a galvo shear unit in front of the camera on an OPSIM instrument. While the details of the OPSIM system have been published elsewhere^12^, in short it uses a Nikon NA 1.35 100× silicone oil primary objective and employs structured light sheets that are tilted by 45° to the optical axis. The structured light sheets are generated in a Michelson interferometer, and a rapid image rotator is used to switch between three azimuthal orientations of the illumination and detection path. The line spacing of the structured light sheets can be tuned but faces an upper limit of about 300 nm for an excitation wavelength of 488 nm, limited by the numerical aperture of the objective and the off-center position of the illumination beams in its pupil.

The newly added shear unit consists of two galvo mirrors (6220H, Novanta Photonics, both equipped with a Y-mirror paddle), orientated orthogonal to each other, in front of the detection camera. The two orthogonal shear directions, which are aligned to the X- and Y-dimensions of the camera, were used to fine align the shear direction to the scan direction of the light sheet.

For a conventional OPM projection, the nine SIM frames were added together. For equidistant phase steps (spanning one period of the pattern), summing the structured illumination images results in an image with uniform illumination, which has been recognized by work of Gustafsson et al.^2^.

### Shear calibration

We used a two-step procedure to ensure correct calibration for the sheer galvo unit. First, we performed a coarse calibration using a micro-ruler. For a top-down view direction, we expect a $\sqrt{2}$ stretch in the projection direction given the 45° tilt angle of the tertiary imaging system. The shear amplitude was adjusted until the micro-ruler image appeared $\sqrt{2}$ stretched in the shear direction. Second, we performed fine calibration using two bead-coated coverslips, sandwiched together, with a ~30 μm gap between the coverslips (See also Supplementary Fig. 2A). We densely coated the bottom coverslip with 100 nm fluorescent nanospheres (ThermoFisher, F8803) and the top coverslip sparsely with 500 nm fluorescent nanospheres (ThermoFisher, F8813). Subsequently, we filled the gap between the two coverslips with water. While imaging the double-layer coverslip sample, the shear factor was empirically varied in small steps.

At each step, we tested if registering the projection images for the three SIM orientations (see also Fig. 1) was successful, i.e., how well the 100 nm and 500 nm beads overlapped (Supplementary Fig. 2B–D). If the shear amplitude was not optimal, i.e., the resulting projection axis was not the same for each SIM orientation, the registration algorithm could not converge on a solution to simultaneously register the beads projected from the bottom and top coverslip (Supplementary Fig. 2C). The requirement for precise physical registration versus post hoc non-rigid computation registration is a key difference between POPSIM and the original OPSIM implementation.

We note that for this manuscript, we have used a home-made bead-sample, where the size of the beads allowed us to identify the bottom and top surface in a projection. More sophisticated fluorescent test targets with matching grids on the top and bottom surfaces could be manufactured by direct laser writing^35^.

### Mammalian cell culture

U2OS cells expressing EGFP-Sec61B to label the ER were a kind gift from Dr. Jens Schmidt. The U2OS cells labeled with GFP-OMP25 were a kind gift from the lab of Jonathan Friedman. U2OS cells labeled for ER were cultured in RPMI (Gibco, A4192301) media supplemented with 10% fetal bovine serum (FBS) and Pen/Strep at 37 °C, 5% CO_2_.

U2OS cells labeled for mitochondria were cultured in DMEM supplemented with 10% FBS (Sigma), 25 mM HEPES, 100 units per ml penicillin and 100 μg ml^−1^ streptomycin.

### Data preprocessing

For the 2D registration of images corresponding to the three illumination directions, the same computational pipeline as in OPSIM was used^12^, but adapted to two dimensions. The images were compressed by a 1/$\sqrt{2}$, rotated by ±60° and coarsely aligned to each other using cross correlation and the *fminsearch* function in MATLAB. After this coarse registration, pairwise multiscale affine registration was applied. To this end, the images were downsampled progresssively in pyramidal fashion at 1×, 2×, 4×, 8×, and 16×. The 4 × 4 affine transformation matrix was then learnt first by aligning data volumes at the coarsest 16× scale. The fitted parameters then initializes the affine matrix for the finer 8x level and so on to the 1× scale.

### SIM reconstruction

We applied the same reconstruction algorithm as in the slice-by-slice processing used in OPSIM^12^. In short, for each illumination direction, a linear equation system is solved in Fourier space on a pixel-by-pixel basis to separate the different information bands (called zero-order band and sidebands here.). The sidebands are then numerically shifted to their corresponding position in reciprocal space. The different bands are added together in reciprocal space, and averaged in areas they overlap. Instead of the traditional Wiener Filtering used in Gustafsson/Heintzmann implementations of SIM, we applied 5–10 iterations of Richardson Lucy deconvolution.

## Supplementary information

Supplementary Information

Supplementary Movie 1

Supplementary Movie 2

## Data Availability

Data underlying the results presented in this paper are available publicly on this repository: https://zenodo.org/record/8277661.

## Code availability

The MATLAB scripts used in this manuscript are available under: https://github.com/AdvancedImagingUTSW/manuscripts/tree/main/2023-POPSIM.
