# Supplementary material for: Projective oblique plane structured illumination microscopy
Source: Npj Imaging. 2023 Nov 28;1:2. doi: 10.1038/s44303-023-00002-2 (PMC12118692; doi:10.1038/s44303-023-00002-2)
Supplement: Supplementary file 1 — Supplementary Information [file 44303_2023_2_MOESM1_ESM.pdf]

Supplementary Information

Projective oblique plane structured illumination microscopy

Authors

Bo-Jui Chang[1], Douglas Shepherd[2], and Reto Fiolka[1]

Affiliation

[1]Lyda Hill Department of Bioinformatics, University of Texas Southwestern Medical Center, Dallas, TX 75390, USA

[2]Center for Biological Physics and Department of Physics, Arizona State University, Tempe, AZ 82587, USA

Correspondence

Correspondence to Reto Fiolka: reto.fiolka@utsouthwestern.edu

Supplementary Notes

Supplementary Note 1 Effects of the projection angle

For POPSIM, physically matching the projection angles for each illumination direction is important to allow 2D registration. This constraint is schematically illustrated in **Supplementary Figure 1**. Shown are two scenarios, a "top-down" projection along the z-axis (**Supplementary Figure 1A**) and one that is projected slightly off from the z-axis (**Supplementary Figure 1B**). As the viewing angles differ in those two cases, objects from different heights (z-position) are mapped to different locations relative to objects at the bottom. When one attempts to map those two projections on top of each other (**Supplementary Figure 1C**), one cannot align all features properly; in the schematic shown, the bottom feature is aligned, but features from higher up will diverge (see also **Supplementary Figure 2**).

We note that anyone who has built an OPSIM can generate "digital" POPSIM data by summing the de-skewed and rotated volumes along the z-axis for each SIM direction. By introducing slightly different views (i.e., a few degrees off from the z-axis), one can illustrate the effects of a suboptimal shear calibration. This could potentially be useful to get an understanding of the effects of a fixed, analog projection.

Supplementary Note 2 Speed comparison to OPSIM

Here we compare the camera limited speed of both OPSIM and POPSIM based on the specifications of the Hamamatsu ORCA Flash 4 camera that has been used in the experiments. We assume a 10-μm tall sample, and an 800x800 pixel wide projection image that is being acquired. With a pixel size of 114 nm, and a $\sqrt{2}$ stretching of the projection image, an effective length of 64 microns is sampled in the scan direction.

Supplementary Figure 3 shows the geometry for either case for one SIM direction. In OPSIM, narrow images with a region of interest (ROI) of 800x128 pixels are acquired to sample the volume. A "height" of 128 pixels is needed to cover a 10-micron tall volume (128 x 0.114 μm / $\sqrt{2}$ =10.3μm). With a step size of 0.32 μm for the OPSIM acquisition, 200 images need to be acquired to form a Nyquist sampled stack. For POPSIM, the entire volume is mapped in one exposure onto an 800x800 pixel image.

Per Hamamatsu's specifications, an image with 128 lines can be acquired with a maximum rate of 579 frames/s. For 800 vertical lines, the maximum rate is 200 frames/s. Note that for the sCMOS camera, the number of horizontal pixels does not affect the overall frame rate. Thus, for this example, the factor by which projection imaging is faster than a 3D stack acquisition is:

(200 / 579) x (200) ~ 69.

Supplementary Note 3 Noise comparison between standard and projection imaging

Here we compare the effect of readout noise for standard 3D stacking and projection imaging in different regimes. This analysis illustrates the differences between OPSIM and POPSIM but is simplified in its scope: a rectangular volume is simulated, which is either 3D imaged in full by light-sheet microscopy or is acquired as a projection. We thus leave out some post processing steps such as data de-skewing which occur in an OPM.

Nevertheless, our model highlights the differences in read-noise when data is numerically projected from a 3D stack, or if an analog projection is formed instead.

We follow a strategy we have previously used for simulated fluorescence microscopy data[1]. Here, we simulated a ground truth volume filled with randomly distributed diffraction limited emitters that each occupy a single voxel on the 3D grid, given by $S(r)$. The 3D grid size of this simulation is over-sampled by a factor of 3 compared to the camera grid (simulation grid spacing = .038 μm; camera grid = .114 μm, the latter corresponding to the pixel sampling used by the experimental setup used in this manuscript).

Simulated emitters were then multiplied by the product of photon emission rate, exposure time, and spatial illumination irradiance, given by $P(r)$. The resulting "emission" object was then convolved with a simulated 3D PSF, $h(r)$, for a light-sheet microscope with NA_{ex}=0.1, NA_{em}=1.35, λ_{ex}=.488 μm, λ_{em}.515 μm [1,2]. Without loss of generality, we here assume uniform illumination when forming $P(r)$.

The fluorescence intensity in the imaging plane is given by

$$I(r) = [P(r) \cdot S(r)] * h(r)$$

where $*$ is the convolution operator.

The camera detects the integrated irradiance over each individual pixel, so we must rewrite the fluorescence intensity in a discrete form,

$$I = Bh[P \odot S]$$

where we regard I , P , and S as vectors and B and h as linear operators. Here $\odot$ represents elementwise multiplication and B is the binning operator that which sums a 3 x 3-pixel area (The binning operator down samples the data onto the camera grid).

For standard imaging, we generate a 3D volume by discrete sampling of individual focal planes every .115 μm. For the pixels in each image, we determine the "noisy" value by drawing from a Poisson distribution with the mean value given by the pixel value I_n^k, where k denotes the axial focus position and n denotes the pixel index. We model the readout noise of an sCMOS camera by assuming a gain, G , of 2 ADU/e$^-$, adding an offset, o , of 100 ADU, and Gaussian noise with standard deviation, σ_R, 1.6 e$^-$ to generate observed data D_n^k,

$$D_n^k = GPoisson\{I_n^k\} + Gaussian(0, \sigma_R) + o.$$

For projection imaging, we first summed the fluorescence intensity $I(r)$ along the axial direction to yield the projected fluorescence intensity. This can be thought of as a projection operator B', that performs the summation in z before binning the pixels in XY,

$$I^{pro} = BB'h[P \odot S].$$

After generating I^{pro}, the camera noise is applied in the same manner as the standard imaging model to generate $D_n^{k\,(pro)}$, with k equal to 1.

Two important differences between standard 3D and projection imaging are 1) the analog summation of photons for each pixel in projection imaging and 2) the accumulation of camera read noise in each frame of the 3D volume for standard imaging, while projection imaging only accumulates a single camera read noise event. We note a more realistic sCMOS camera simulation is possible by calibrating the per-pixel noise using the methods outlined in Huang et al[3]

To compare camera limited and sampled limited cases, we changed the exposure times and photon emission rates accordingly.

For camera limited simulations, we utilized the exposure times discussed in **Supplementary Note 2**. Specifically, 1.7 ms per plane for standard imaging and 5 ms per image for projection imaging. We hand tuned the photon emission rate to obtain a low signal case (~10 recorded photons/emitter) and moderate signal case (~100 recorded photons/emitter) in each standard imaging plane. We find a moderate increase in contrast in the projection image for the low photon count case and nearly identical results for the moderate photon count case (**Supplementary Figure 4A-B**). Because the projection imaging rate is roughly 69X faster, this suggests for camera limited applications the projection mode may be useful for imaging fast biological dynamics where 3D information is not critical.

For sample limited simulations, we utilized 2 ms per plane for standard imaging and 32 ms per plane for the projection imaging. For the simulations presented here, this still represents a 4x increase in imaging speed in projection mode. We again hand tuned the photon emission rate to a low signal case (~10 recorded photons/emitter in the projection image) for the projection imaging case. Due to the accumulation of photons at a given axial position and relative lack of camera read noise, signal begins to emerge in the projection imaging case at lower photon emission rates than standard imaging (**Supplementary Figure 4C**).

Finally, we simulated a regime where the total number of photons accumulated, before accounting for read noise, is the same across the entire standard 3D volume and single projection image (**Supplementary Figure 4D**). In other words, the same sample irradiance occurs for either mode. Here, we find a moderate increase in contrast in the projection mode, again due to the lack of additive read noise.

The simulations are implemented in Python. An interactive *Google Colaboratory* version is hosted under this repository: https://github.com/QI2lab/ProjectionSimulation

Supplementary Figures

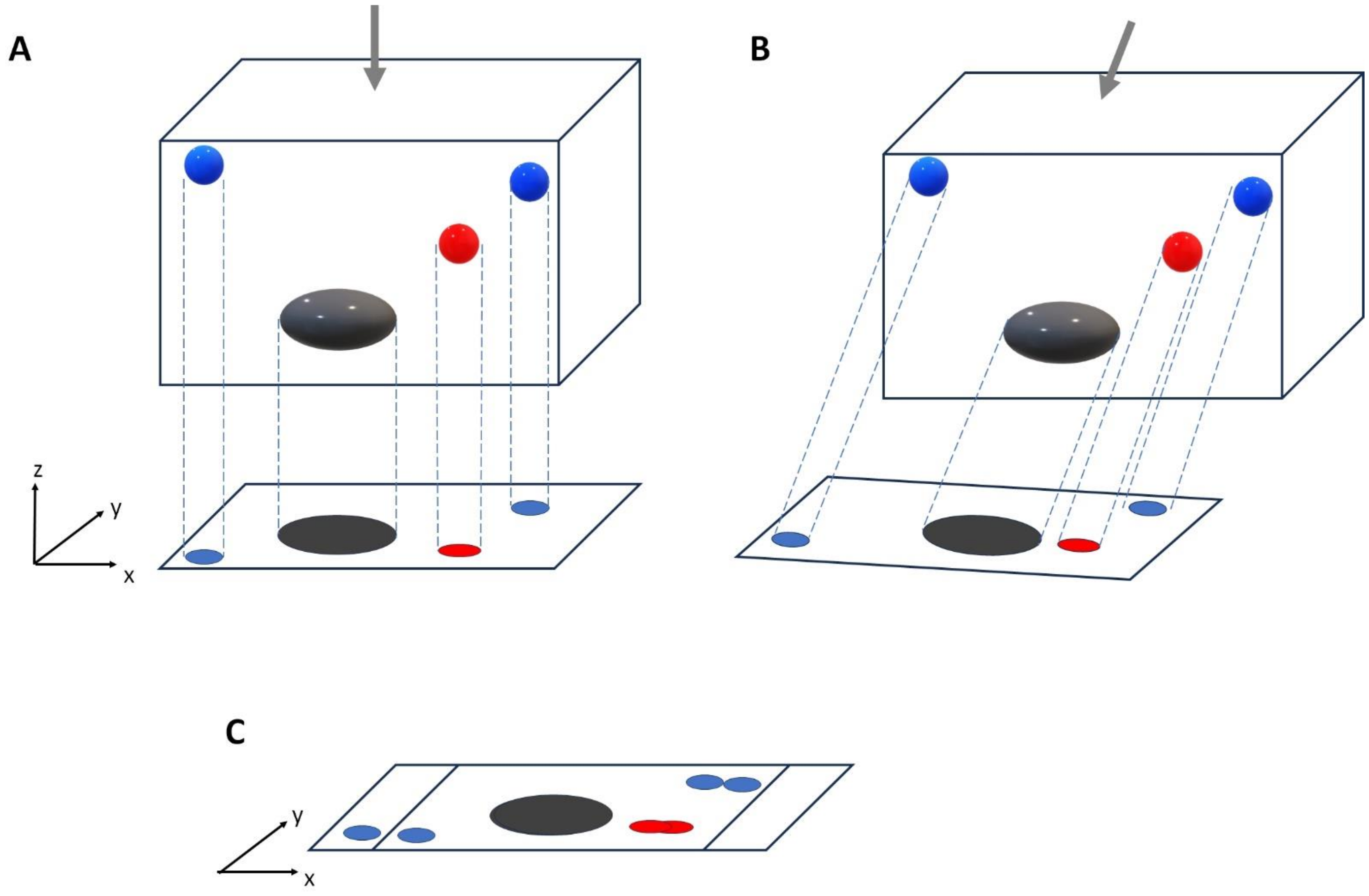

Supplementary Figure 1 – Effect of projection angle on registration of images. A Schematic depiction of a projection along the z-axis of a volume containing three spheres (red and blue) and one ellipsoid (gray). Gray arrow on top denotes the projection direction. **B** A projection of the same volume, but under an angle slightly off from the z-axis. **C** Schematic depiction when registering (or aligning) the two projections from **A** and **B** on top of each other.

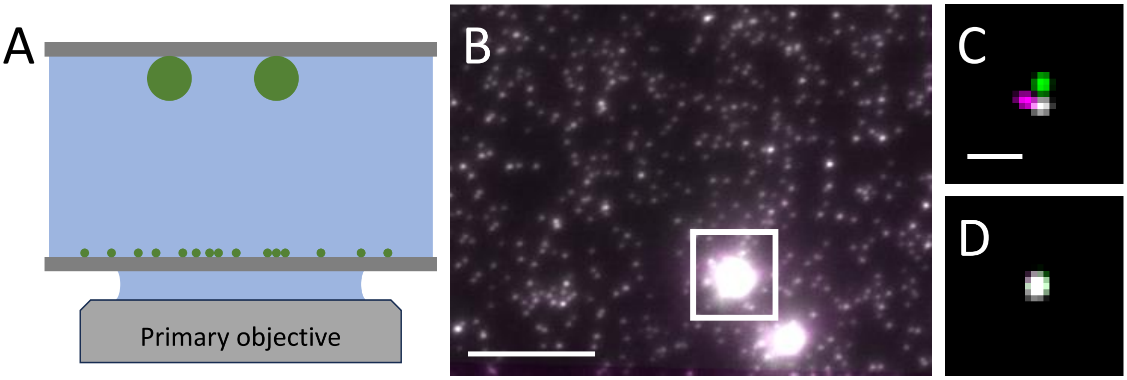

Supplementary Figure 2 - Calibration of the projection viewing angles by mapping two bead layers on top of each other. A Schematic illustration of two opposing coverslips, one densely coated with 100nm fluorescent nanospheres on the bottom and one sparsely coated with 500nm nanospheres on top. **B** Overlay of three registered projections corresponding to the SIM orientations 1-3 (color coded green, magenta and gray). The two bright spots are two 500nm beads which are saturated to better show the overlap of the 100nm beads. C Inset of bright 500nm nanosphere in the white rectangle in **A**, with contrast adjusted. D the same sphere after calibration of the shear parameters. Scale bars: **A**: 10 µm; **B**: 2 µm.

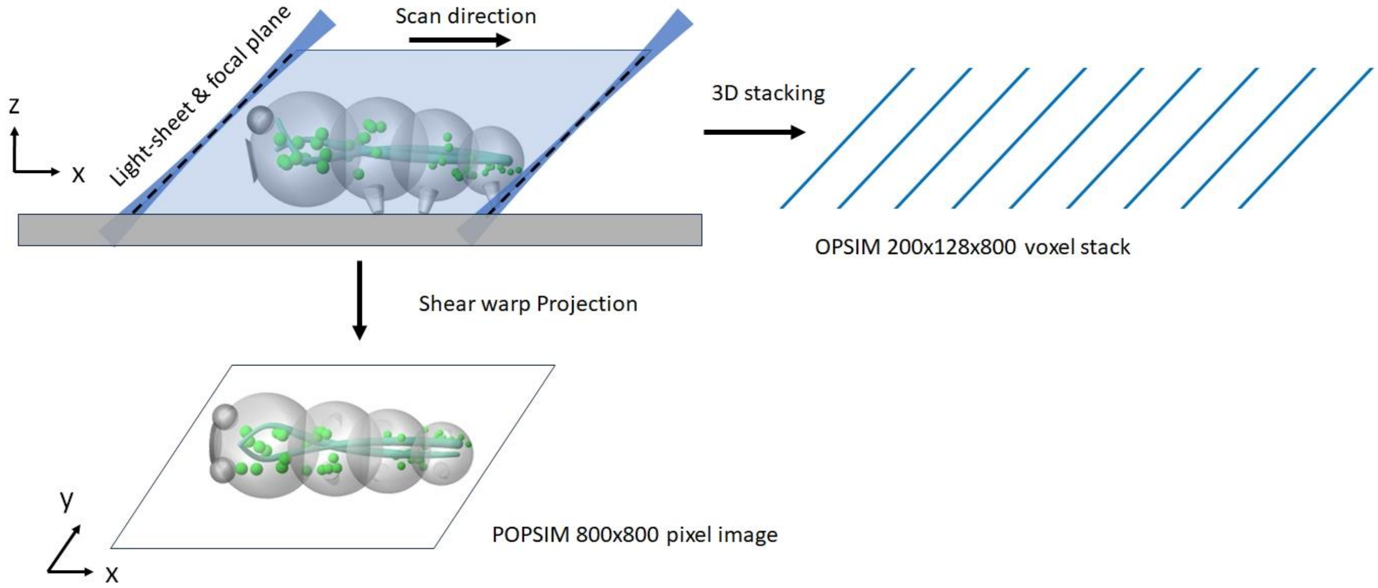

Supplementary Figure 3 –Comparison of OPSIM and POPSIM acquisition. Schematic depiction of a volume (light-blue parallelogram) that is being sampled with the light-sheet and corresponding focal plane. For OPSIM, a 3D stack of a focal series is acquired (right, individual blue lines illustrate images in the stack). Below, in one POPSIM acquisition, one projection image of the light-blue shaded volume is acquired.

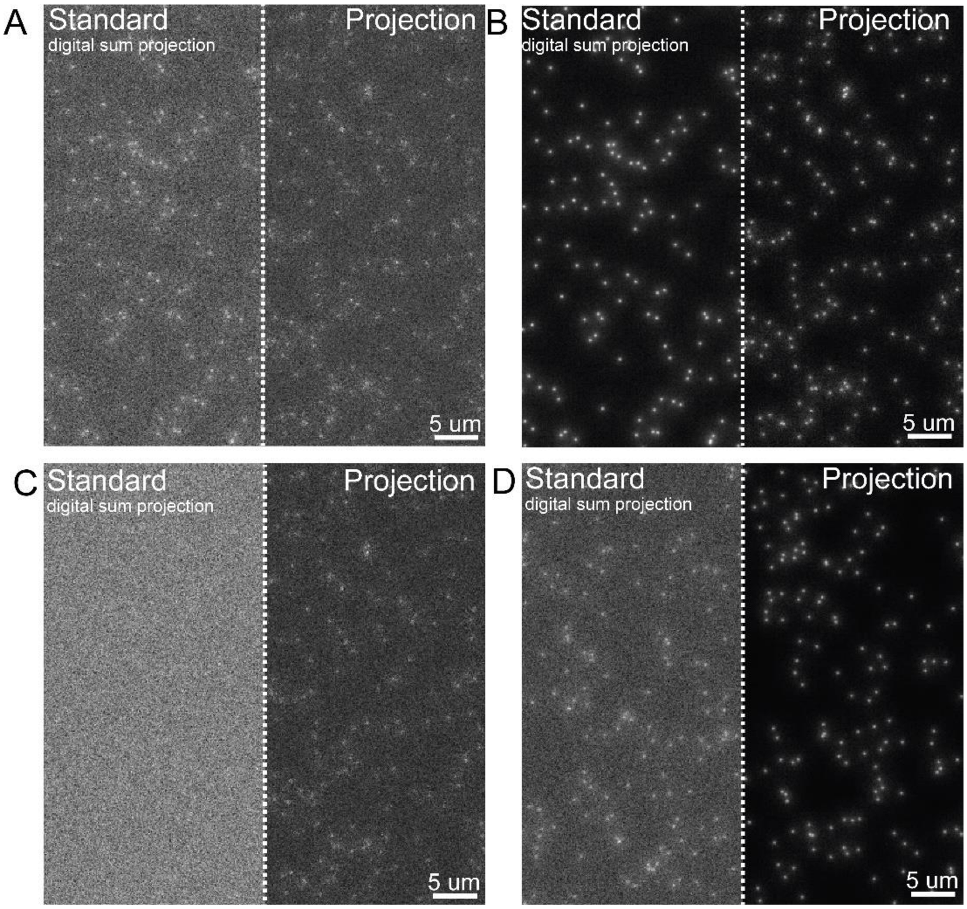

Supplementary Figure 4 –Comparison of noise in standard 3D imaging and projection imaging. A. Camera limited case #1. Low photon count simulation (~10 photons/emitter) for 1.7 ms per plane standard 3D imaging and 5 ms total exposure projection imaging. The ratio of projection to standard 3D imaging speed is 69. **B. Camera limited case #2.** Moderate photon count (~100 photons/emitter) for 1.7 ms per plane standard 3D imaging and 5 ms total exposure projection imaging. The ratio of projection to standard 3D imaging speed is 69. **C. Sample limited case.** Low photon count simulation (~10 photons/emitter) for 2 ms per plane standard 3D imaging and 50 ms total exposure projection imaging. The ratio of projection to standard 3D imaging speed is 4. **D. Equal photons case.** ~38,000 total photons (before noise) spread a standard 3D volume or projection image. The ratio of Projection to standard 3D imaging speed is 1.

Supplementary References

1 Brown, P. T., Kruithoff, R., Seedorf, G. J. & Shepherd, D. P. Multicolor structured illumination microscopy and quantitative control of polychromatic light with a digital micromirror device. *Biomedical Optics Express* **12**, 3700-3716 (2021).

2 Lambert, T. https://github.com/tlambert03/psfmodels.

3 Huang, F. *et al.* Video-rate nanoscopy using sCMOS camera–specific single-molecule localization algorithms. *Nature methods* **10**, 653-658 (2013).
